# Biofilm Structures in a Mono-Associated Mouse Model of *Clostridium difficile* Infection

**DOI:** 10.3389/fmicb.2017.02086

**Published:** 2017-10-25

**Authors:** Anna P. Soavelomandroso, Françoise Gaudin, Sandra Hoys, Valérie Nicolas, Gayatri Vedantam, Claire Janoir, Sylvie Bouttier

**Affiliations:** ^1^EA4043, Unité Bactéries Pathogènes et Santé (UBaPS), Univ. Paris-Sud, Université Paris-Saclay, Châtenay-Malabry, France; ^2^Institut Paris Saclay d’Innovation Thérapeutique (IPSIT), UMS IPSIT Université Paris-Sud – US 31 INSERM – UMS 3679 CNRS, Plateforme d’Histologie souris Immunopathologie de Clamart – PHIC, Clamart, France; ^3^Institut Paris Saclay d’Innovation Thérapeutique (IPSIT), UMS IPSIT Université Paris-Sud – US 31 INSERM – UMS 3679 CNRS, Plateforme d’Imagerie cellulaire – MIPSIT, Châtenay-Malabry, France; ^4^School of Animal and Comparative Biomedical Sciences, University of Arizona, Tucson, AZ, United States

**Keywords:** *C. difficile*, biofilm, mono-associated mouse model, colonization, gut, mucus, immunochemistry, CLSM

## Abstract

*Clostridium difficile* infection (CDI) is a major healthcare-associated disease with high recurrence rates. Host colonization is critical for the infectious process, both in first episodes and in recurrent disease, with biofilm formation playing a key role. The ability of *C. difficile* to form a biofilm on abiotic surfaces is established, but has not yet been confirmed in the intestinal tract. Here, four different isolates of *C. difficile*, which are *in vitro* biofilm producers, were studied for their ability to colonize germ-free mice. The level of colonization achieved was similar for all isolates in the different parts of the murine gastrointestinal tract, but pathogen burden was higher in the cecum and colon. Confocal laser scanning microscopy revealed that *C. difficile* bacteria were distributed heterogeneously over the intestinal tissue, without contact with epithelial cells. The R20291 strain, which belongs to the Ribotype 027 lineage, displayed a unique behavior compared to the other strains by forming numerous aggregates. By immunochemistry analyses, we showed that bacteria were localized inside and outside the mucus layer, irrespective of the strains tested. Most bacteria were entrapped in 3-D structures overlaying the mucus layer. For the R20291 strain, the cell-wall associated polysaccharide PS-II was detected in large amounts in the 3-D structure. As this component has been detected in the extrapolymeric matrix of *in vitro C. difficile* biofilms, our data suggest strongly that at least the R20291 strain is organized in the mono-associated mouse model in glycan-rich biofilm architecture, which sustainably maintains bacteria outside the mucus layer.

## Introduction

The obligate anaerobe *Clostridium difficile* is the leading cause of healthcare-associated diarrhea. *C. difficile* infection (CDI) represents 15–25% of antibiotic-associated diarrhea and clinical symptoms range from mild diarrhea to life-threatening pseudomembranous colitis (Evans and Safdar, 2015).

CDI usually appears after ingestion of spores, which are the contaminating forms of *C. difficile.* Concomitantly, the disruption of the intestinal microbiota due to antibiotic therapy supports germination of spores as well as colonization of outgrowing vegetative cells. Vegetative cells express different factors involved in virulence such as several colonization factors and two main virulence factors: the TcdA and TcdB toxins that lead to actin cytoskeleton disorganization and cell death (Janoir, 2016). A third toxin called the binary toxin (CdtAB) has been found in 23% of toxigenic strains in Europe in 2008 (Gerding et al., 2014). It is assumed that CDT potentiates the toxicity of TcdA and TcdB.

Recurrence is a major challenge encountered in the management of CDI, since as many as 35% of patients may undergo a recurrence after the first episode, and this rate increases after further episodes (Marsh et al., 2012). Recurrence corresponds either to a relapse with the same strain or to a re-infection with another strain, which occurs in 38–56% of cases (Barbut et al., 2000). The causes of relapses are not clearly determined, but undoubtedly involve host and bacterial factors. One of the main bacterial features involved in relapsing disease is the ability of *C. difficile* to re-sporulate and persist in the gastrointestinal tract, resisting antibiotic exposure (Deakin et al., 2012). Discrepant results have been reported in the correlation between recurrences and virulence factors of bacteria. Stewart et al. (2013) found that the binary toxin is a predictor of recurrent infection. In contrast, a recent study demonstrated that the outcome of CDI correlates neither with the virulence features nor with the *in vitro* level of sporulation (Plaza-Garrido et al., 2015). Another hypothesis is that relapses may be associated with the persistence of *C. difficile* in a biofilm. Indeed, it is well known that mono- or polymicrobial biofilm formation contributes to chronicity of several infections, such as chronic wounds, cystic fibrosis and periodontitis (Mihai et al., 2015). The persistence of bacteria in the host is supported by biophysical characteristics of biofilms, which result in avoidance of host immunity and resistance to environmental stresses, such as antibiotic therapy. Biofilm is defined in *stricto sensu* by a 3-D organization of a bacterial community adherent to an abiotic or a biotic support, and embedded in an extracellular polymeric substance (Costerton et al., 1978). In addition to its role in chronic infections, biofilms may participate in colonization of both commensal and pathogenic bacteria. Asymptomatic carriage of *C. difficile* has been described, and is now recognized as a reservoir for *C. difficile* transmission (Donskey et al., 2015), and *C. difficile* biofilm could also be involved in asymptomatic carriage.

The ability of *C. difficile* to form a biofilm *in vitro* was first described 5 years ago (Donelli et al., 2012). Since then, the biofilm-producing phenotype of *C. difficile* has been confirmed, and several factors that modulate the biofilm formation have been identified. In particular, we have previously shown that the cell wall-associated cysteine protease Cwp84, involved in the S-layer maturation, negatively modulates biofilm formation by a still unknown mechanism (Pantaléon et al., 2015). In contrast, the regulator of quorum sensing LuxS and the key regulator of sporulation Spo0A positively modulate biofilm formation (Dawson et al., 2012; Đapa et al., 2013). As observed for other species, the extrapolymeric matrix of *C. difficile in vitro* biofilm is composed of polysaccharides including cell wall-associated PSII, proteins and DNA (Đapa et al., 2013; Semenyuk et al., 2014; Pantaléon et al., 2015). The relationship between the ability of *C. difficile* to form a biofilm and its capacity to colonize and to persist in a host is not yet known. Moreover, how *C. difficile* associates with biotic surfaces is still poorly understood. Lawley et al. (2009) reported individual or grouped *C. difficile* cells observed closely associated with damaged epithelia in conventional mice. A recent work suggests that in the conventional murine model of CDI, *C. difficile* is a minority member of the microbial communities during infection and is associated with the outer mucus layer (Semenyuk et al., 2015). *C. difficile* was also shown to associate to polymicrobial biofilms in an *in vitro* human chemostat gut model particularly in spore form, while planktonic bacteria were primarily vegetative cells (Crowther et al., 2014a,b).

To date, there are no data on the ability of *C. difficile* to form a mono-microbial biofilm *in vivo*. The aim of our study was therefore to investigate the level of colonization achieved by different *in vitro* biofilm-producing *C. difficile* strains in the intestinal tract of germ-free mice, and define the spatial organization of bacteria associated with gut tissues. The *C. difficile* mono-associated mouse model was chosen because this is a simplified model of colonization, devoid of competitive interaction; in addition this is an easy tool for the observation of biofilm structure.

## Materials and Methods

### Bacterial Strains and Media

The *C. difficile* strains used in this study are described in **Table 1**. Bacteria were grown at 37°C, under anaerobic conditions (90% N_2_, 5% CO_2_ and 5% H_2_), in fresh BHISG (Brain Heart infusion broth [Difco, United States] supplemented with 1.8% Glucose, 0.1% L-Cysteine and 0.5% yeast extract) (Pantaléon et al., 2015). This sugar-rich medium is commonly used for *C. difficile* in *in vitro* biofilm assays (Dawson et al., 2012; Đapa et al., 2013; Semenyuk et al., 2014) because the presence of glucose in the culture is essential for optimal biofilm formation (Đapa et al., 2013).

**Table 1:** Characteristics of *Clostridium difficile* strains used in this study.

*Clostridium difficile* strains	Ribotype	Toxinotype	Origin
R20291	RT 027	III	Stoke Mandeville epidemic 027 strain (United Kingdom)
P30	RT 014/020	0	Strain isolated from poultry
630Δerm	RT 012	0	Erythromycin sensitive derivative of the 630 strain (Switzerland)
630Δ*erm cwp84::erm* (named thereafter *cwp84* mutant)	RT 012	0	Mutant strain derived from the 630Δerm (Clostron)

### *In Vitro* Biofilm

Biofilm assays were performed in 24-well polystyrene plates (Costar, United States). Overnight suspensions of each *C. difficile* strain in BHISG broth were diluted in fresh BHISG and 10^6^ bacteria were added to each well. Plates were incubated at 37°C under anaerobic conditions. After 72 h of incubation, the supernatant was removed carefully, and the wells were rinsed twice with PBS. The biofilm biomass was thereafter quantified by crystal violet staining, and enumeration of viable cells. Crystal violet (ACROS Organics^TM^, United States) quantification was performed as previously described (Dawson et al., 2012; Đapa et al., 2013; Pantaléon et al., 2015). For viable cell enumeration, 1 ml of sterile pre-reduced PBS was added to each well, the biofilm formed in the bottom was scraped, diluted and plated on BHI agar supplemented with 3% defibrinated horse blood. For each quantification method, experiments were done on two wells from the same plate, and biological replicates were performed at least three times.

Biofilm architecture was then analyzed by confocal laser scanning microscopy (CLSM). Prior to aerobic transfer, the plate was covered with parafilm to minimize oxygen exposure. The biofilm was stained 3 days post-incubation with the LIVE/DEAD^®^ *Bac*Light^TM^ Bacterial Viability Kit (Thermo Fisher Scientific, United States): 200 μl of the diluted mixture (1:1000) was added per well and incubated 15 min at 37°C under anaerobic conditions. Samples were visualized with a LSM 510 microscope (Carl Zeiss Inc., Germany). SYTO^®^ 9 and propidium iodide exhibit an excitation at 483 and 535 nm and a fluorescence emission at 503 and 617 nm, respectively. Horizontal plane images with a z-step of 0.98 μm were acquired for each strain at three different areas in each well. The thickness were determined directly from the confocal stack images using the software image J.

The Mann–Whitney *U* Test was used for statistical analyses. *P* < 0.05 was considered statistically significant.

### Animal Model

Six to 8 week-old germ-free C3H/HeN female mice were purchased from CDTA (CNRS Orléans, France). All animal experiments were performed according to European Union guidelines for the handling of laboratory animals and all procedures were approved by the Ethics Committee CAPSUD (Protocol 2012-109). Mice were housed in sterile isolators with *ad libitum* access to food and water. Before experiments, each animal was confirmed to be germ-free by Gram staining of feces and by inoculating feces into BHI broth and incubating the broth for 48 h, either aerobically or anaerobically. Mice were challenged by oral gavage with 10^6^ bacteria in 0.5 ml volume. This inoculum was prepared as follows: an overnight culture in BHISG was pelleted, washed twice with PBS, and then re-suspended in PBS to a final concentration of approximately 2 × 10^6^ vegetative cells/ml, estimated by microscopic cell counting. This bacterial concentration was checked thereafter by enumerating both vegetative cells and spores. Seven days post-infection, mice were sacrificed and different parts of the intestinal tract (jejunum, ileum, cecum, and colon) as well as fecal samples were collected, either for enumeration of bacteria, confocal microscopy analyses or immunohistochemistry analyses.

### Intestinal Colonization Levels

To determine the mean level of colonization of the four *C. difficile* strains, enumeration of bacteria in the digestive tract of mono-associated mice was performed from three mice for each strain tested. First, the contents of the different intestinal parts were collected and used for enumeration of luminal bacteria (LB). Second, after three PBS rinses, the mucosal tissues were homogenized for 1 min with Ultra-Turrax T25 (IKA^®^, Labortechnik, Germany), and tissue-associated bacteria (TAB) enumerated. Both vegetative cells and spores were enumerated in all samples. Vegetative cells were counted by plating serial dilutions onto BHI agar supplemented with 3% horse blood. Then, samples were treated with ethanol, as previously described (Pantaléon et al., 2015) and spores were counted by plating serial dilutions onto BHI agar supplemented with 3% horse blood and 0.1% taurocholate (Sigma, United States).

### Confocal Laser Scanning Microscopy (CLSM)

The spatial organization of tissue-associated bacteria was determined by CLSM analysis of mouse mucosa from at least three mice for each strain. Seven days post-infection, the different parts of the intestine were collected (jejunum, ileum, cecum, and colon). After removal of intestinal content, the tissues were washed three times in 10 ml of PBS, spread on a glass slide and stained with the LIVE/DEAD^®^ *Bac*Light^TM^ Bacterial Viability Kit. Samples were visualized as described above with the same parameters defined as for *in vitro* assay. During the *Z*-stack acquisition, an average of five areas on tissue sample was analyzed. In addition, three-dimensional projections were reconstructed from *x* to *z* stacks using the software Imaris (Bitplane, United Kingdom).

### Immunohistochemistry Analyses

Immunochemistry was performed on three mice orally challenged with either the R20291, the P30 or the 630Δ*erm* strain. As a negative control, a germ-free mouse was labeled similarly. Cecum and colon were sampled after the sacrifice of mice, fixed in Carnoy’s solution and paraffin sections (7 μm) obtained.

Bacterial staining was performed with two fluorescent dyes that stain both eukaryotic and prokaryotic double-stranded DNA. Samples were stained either with SYTO^®^ 9 at 1:1,000 dilution, or Hoechst at 1:500 dilution, and incubated 1 h at 37°C, before washing with PBS.

For mucus straining, rabbit antisera against Mucin 2 (H-300):sc-15334 (anti-Muc2, Santa Cruz Biotechnology, Inc.) were used at 1:100 dilution for 1 h at room temperature. After washing, the sections were incubated with a secondary antibody for 1 h at room temperature before detection. Two different secondary antibodies were used, depending on the objective of the labeling. For detection of mucus only, we used a biotinylated antibody that was detected with streptavidin-HRP complex followed by 3,3′-diaminobenzidine or 3-amino-9-ethylcarbazole detection (LSAB kit, Dako, United States); sections were then counterstained with hematoxylin. In some experiments, we performed a double-staining of mucus and bacteria to study the localization of bacteria with respect to mucus. In this case, we used Alexa Fluor 594 secondary antibody (A-11007, Life Technologies, United States), detected by immunofluorescence. A slide stained only with the secondary antibody was used as an additional control for specificity of the staining.

In a final set of experiments, the bacterial polysaccharide PS-II was stained by immunochemistry using rabbit antibodies against PS-II at 1:1000 dilution for 1 h at room temperature. The sections were washed and incubated with a secondary antibody Alexa Fluor 594 for 1 h at room temperature before detection.

Slides were scanned by the digital slide scanner NanoZoomer 2.0-RS (Hamamatsu, Japan), which allowed an overall view of the samples. Images were digitally captured from the scan slides using the NDP.view2 software (Hamamatsu).

## Results

### Biofilm Formation Varies between *C. difficile* Isolate *in Vitro*

We questioned whether the ability of strains to form a biofilm could predict their ability to colonize the gut. We studied four strains that were chosen according to their ability to form a biofilm on abiotic surfaces, and their level of colonization was investigated in the mono-associated mouse model.

Using crystal violet staining, we were able to classify the strains in three categories that display significant differences in their biofilm-producing abilities (Supplementary Figure S1A): one high-biofilm former (*cwp84* mutant) (mean absorbance > 15), two moderate-biofilm formers (R20291 and P30) (5 < mean absorbance < 15) and one low-biofilm former (mean absorbance < 5) (630Δ*erm*). Overall, the biomass quantification by crystal violet staining was proportional to the viable cell count (Supplementary Figure S1B). By confocal microscopy analysis (Supplementary Figure S1C) we confirmed the ability of the *cwp84* mutant to form the most significant biofilm, as illustrated by the mean thickness of each biofilm (Supplementary Figure S1D). In a previous study (Pantaléon et al., 2015), we showed that the surface of the *cwp84* mutant was more hydrophobic than the parental strain but the initial adhesion was not altered. Therefore, the increased biofilm is not related to an increase in adhesion in the early stages.

### *C. difficile* Colonization Burden of the Germ-Free Murine Intestine Is Location-Specific

The ability to colonize the intestine of germ-free mice was studied for each of the strains described in **Table 1**. Bacterial colonization was determined by the amount of both luminal (LB) and tissue-associated bacteria (TAB); vegetative cells and spores were enumerated.

**FIGURE 1:**
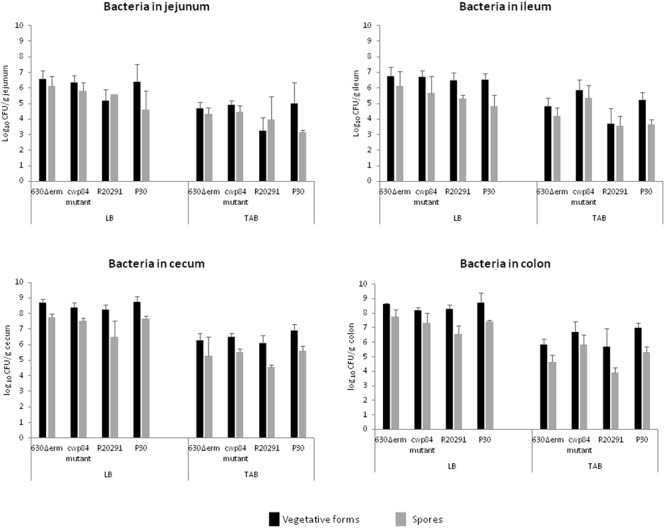
Intestinal colonization of monoxenic mice by *Clostridium difficile* strains. Quantitation of viable vegetative cells (luminal bacteria, LB and tissue-associated bacteria, TAB) in the different parts of the intestine (jejunum, ileum, cecum, and colon). The data represent the average of three independent assays. No statistical analysis was performed because the number of mice tested was limited to three per strain.

Overall, titers of luminal bacteria of all four *C. difficile* strains were similar, and indistinguishable in the different parts of the gut (jejunum, ileum, cecum, and colon) at 7 days post-infection (**Figure 1**). However, overall colonization levels were 100-fold lower in the jejunum and ileum as compared to the cecum and colon (**Figure 1**). We compared the amount of adherent bacteria in the different parts of the gut. Titers of tissue-associated bacteria were slightly different according to strains in all parts of the gut and except for the ileum, the strains could be classified as follows: P30 > cwp84 mutant > 630Δerm > R20291. Gram staining was performed on bacterial suspension after homogenization and vortexing to ensure that bacteria no longer formed clusters.

Luminal bacteria and TAB spores were present in smaller amounts as compared to vegetative cells and represented less than 15% of total population, except for the R20291 strain in jejunum. Surprisingly, in cecum and colon, the ratio of spores/vegetative cells seemed to be lower for R20291 strain compared to the P30 and the *cwp84* mutant strains.

### Distribution of Bacteria Over Epithelial Tissues

**FIGURE 2:**
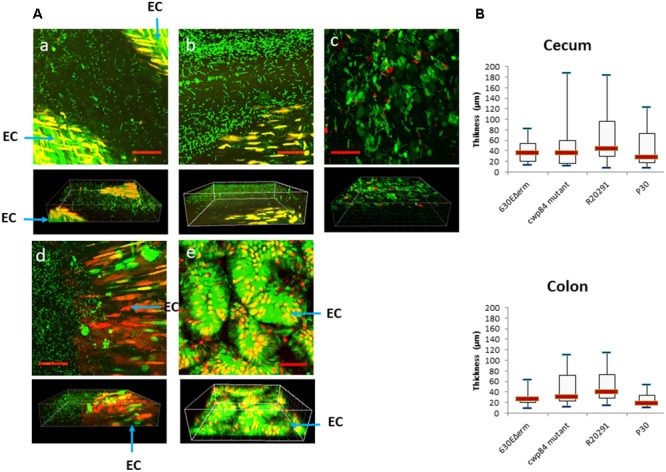
Heterogeneous distribution of bacteria over the tissue in a mono-associated mouse model. Images are representative of at least five fields. **(A)** Confocal laser-scanning microscopy (CLSM) images (*Z*-stacks (above) and 3-D projection (below)] of tissue-associated bacteria obtained from cecum for the 630Δ*erm* (a), the *cwp84* mutant (b), the R20291 (c), the P30 (d) strains and the germ-free mouse (e). Live cells [bacterial (rod) or epithelial] are labeled in green, dead cells are labeled in red. EC, epithelial cells. Scale bars (red): 50 μm. **(B)** Thickness of bacterial 3-D structure in cecum and colon. The thickness of the bacterial 3-D structure is defined by the height on which bacteria are distributed. The thickness was determined directly from confocal *Z*-stack images. At least three mice were used for CLSM analyses for each strain, and at least eight fields per sample were observed. Data are presented as boxplots with median and minimum-maximum whiskers. No significant difference was observed between strains (Mann–Whitney test).

In accordance to the very low amount of bacteria associated with the mucosa in jejunum and ileum (10^3^–10^6^ CFU/g of tissue), we rarely detected bacteria in these tissues by CLSM (data not shown) in contrast to what was observed in the cecum (**Figure 2A**) and the colon (data not shown). In both cecum and colon, irrespective of strain, we observed areas without and with bacteria associated with tissues. When present, bacteria were observed both in cecum and colon, but the bacterial distribution was different according to strain. While bacterial distribution for the P30, 630Δerm and cwp84 mutant was mostly as single cells, the R20291 was organized as bacterial aggregates (**Figure 2A**, Panel c).

Therefore, as observed by 3-D visualization (**Figure 2A**, projections at the bottom), bacteria were organized at the epithelial surface as a 3-D structure, and were never in direct contact with the epithelial cells. With the four strains, we found randomly distributed areas either with a high or a low thickness of the *C. difficile* community and this was particularly observed for R20291 and cwp84 mutant strains. Therefore, no significant difference in mean thickness of the 3-D structure was observed between strains in the cecum or colon (**Figure 2B**).

This organization of *C. difficile* as a bacterial community overlaying the mucosa without contact with epithelium cells was confirmed by Hoechst staining of histological sections of mouse cecum and colon for the R20291 strain (Supplementary Figure S2).

### Murine *C. difficile* Communities Are Associated with Glycopolymers and Mucus

The establishment of the *C. difficile* community as a 3-D structure overlaying epithelial cells in rinsed mucosal tissues could correspond either to bacteria entrapped in the mucus layer, or outside the mucus layer and maintained entrapped by another unknown structure. To address this question, we analyzed the localization of bacteria with respect to the mucus layer.

Mucus was detected in goblet cells, as well as outside of the epithelial cells (Supplementary Figure S4A). In the colon, two distinct layers were clearly detected: the inner layer, which is firmly adherent to the intestinal tissue and the outer layer which is looser and thicker. In both infected and non-infected animals, twice the numbers of goblet cells were detected in the colon as compared with the cecum (Supplementary Figure S4B). The uneven surface of mucus layer could be partially explained by tissue treatment including three rinses. As shown for R20291 strain, villi were more developed and more mature in mice infected by *C. difficile* than in germ-free mice (Supplementary Figure S4A).

**FIGURE 3:**
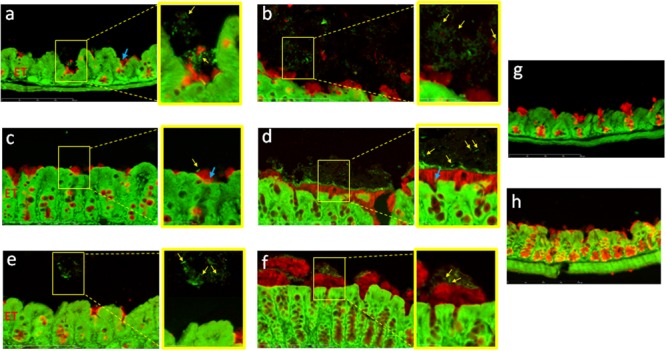
*Clostridium difficile* cells are mainly localized at the surface of the mucus layer. Immunodetection of mucus and fluorescent labeling of bacteria by SYTO^®^ 9 in the cecum **(a,c,e,g)** and in the colon **(b,d,f,h)** of mice infected with strains 630Δ*erm* **(a,b)**, R20291 **(c,d)**, P30 **(e,f)**, or in axenic mouse **(g,h)**. Mucus is stained in red and DNA (bacterial, eukaryotic intracellular and, extracellular DNA) is stained in green. The right panel is the enlarged section of the yellow boxed portion of the image. Yellow arrows indicate position of bacteria outside the mucus layer. Blue arrows indicate position of bacteria in the outer layer of mucus. Scale bar is 200 μm.

Histological sections were further double-stained with SYTO^®^ 9, which labels bacterial, eukaryotic and extracellular double-stranded DNA, and with anti-Muc2 for immunodetection of mucus. A few bacteria were observed on these sections: some of them were embedded in the mucus (**Figure 3** and Supplementary Figure S3B), but most of them were localized outside of the mucus layer, at the interface of the mucus and lumen (**Figure 3**). It is important to note that these structures were observed after three rinses of the cecal and colonic mucosa, suggesting that bacteria were firmly associated with the mucus. However, some of them were present as individual cells, but a majority were entrapped in a defined 3-D structure. Similar images were observed for the R20291, the 630Δerm and the P30 strains (**Figures 3a–f**). In contrast, we did not observe similar structures in the axenic mouse (**Figures 3g,h**).

**FIGURE 4:**
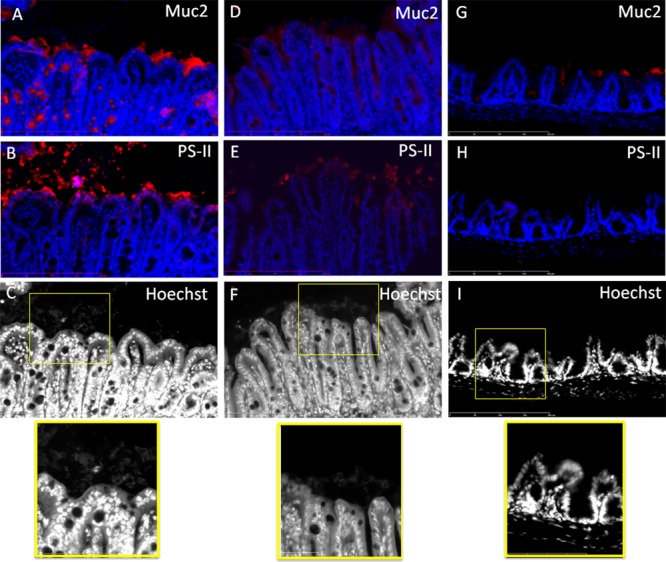
Bacteria localized outside the mucus are embedded in a PS-II matrix in the cecum of R20291-infected mice. Comparison of stained serial sections **(A–C) (D–F) (G–I)**. **(A,B)** and **(D,E)** Represent two different areas of the cecum of mouse infected by the R20291 strain. **(G,H)** Are serial sections from germ-free mouse. Mucus and PS-II are stained in red and DNA (bacteria and epithelial tissue, extracellular DNA) are stained in blue or gray. The bottom panel is the enlarged section of the yellow boxed portion of the image. Scale bar is 200 μm.

**FIGURE 5:**
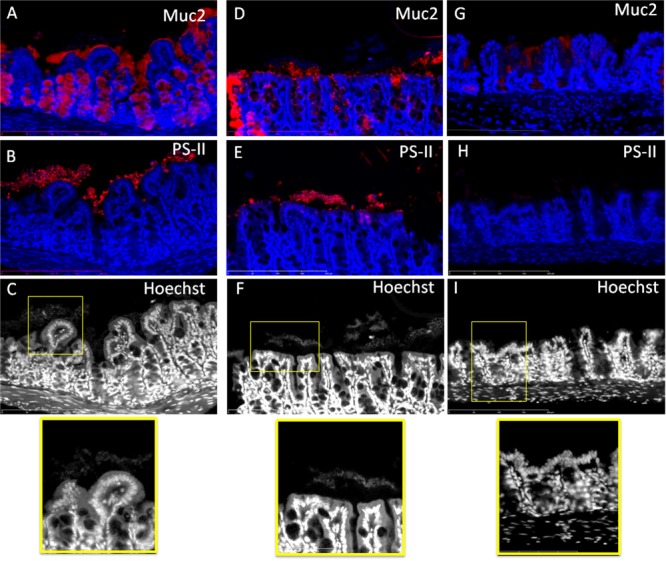
Bacteria localized outside the mucus are embedded in a PSII matrix in the colon of R20291-infected mice. Comparison of stained serial sections **(A–C) (D–F) (G–I)**. **(A,B)** and **(D,E)** Represent two different areas of the colon of mouse infected by the R20291 strain. **(G,H)** Are serial sections from germ-free mouse. Mucus and PS-II are stained in red and DNA (bacteria and epithelial tissue, extracellular DNA) are stained in blue or gray. The bottom panel is the enlargement of the yellow boxed portion of the image. Scale bar is 200 μm.

In order to determine the nature of the structure in which bacteria seem to be entrapped, we performed double staining of two serial sections, one with anti-Muc2/Hoechst and the second one with anti-PS-II/Hoechst. This was performed on cecal and colonic sections of animals infected with the R20291 strain (**Figures 4, 5**). Visualization of multiple areas of the sections in **Figure 4** (cecum) and **Figure 5** (colon), confirmed that bacteria were not in contact with the epithelial cells, and that they were separated by the mucus layer lining the epithelium tissue. However, some differences were observed between cecum and colon. The cecal sections observed showed that bacteria were distributed in equivalent manner in and outside the mucus and that PS-II labeling was observed accordingly (**Figure 4**). In the colon, comparison of images of serial sections (A and B compared with D and E) revealed that dense islets of bacterial PS-II overlayed the mucus layer, and that bacteria were mainly entrapped in these islets. PS-II staining was observed only outside the mucus layer. As showed in Supplementary Figure S3, bacteria inside the mucus were not stained significantly by the PS-II, suggesting that islets of PS-II may correspond to accumulation of extracellular PS-II. Noticeably, no structure corresponding to PS-II was observed lining the mucosal gut both in cecum and in colon in the axenic mouse.

## Discussion

*Clostridium difficile* is able to colonize the human colonic niche upon dysbiosis, a process that could result in asymptomatic carriage, infection, or persistence of bacteria post-infection despite treatment (leading to disease relapse). For several pathogenic bacteria, primary colonization and persistence in the host has been correlated with biofilm formation (Cooper et al., 2014; Mihai et al., 2015; Lund-Palau et al., 2016). *C. difficile* has been shown to form biofilms on abiotic surfaces, but the role of a sessile lifestyle in the colonization of human gut has not yet been addressed. Moreover, there is no evidence to date that *C. difficile* is able to form a biofilm *in vivo*, although this bacterium has been found associated within the mucus in a polymicrobial community in a conventional mouse model of *C. difficile* infection (Semenyuk et al., 2015). Intestinal microbiota have been described by some authors as a biofilm, and in that case *C. difficile* may be recruited into this poly-microbial community (Palestrant et al., 2004; Macfarlane and Dillon, 2007). However, there is still extensive debate on whether the intestinal microbiota is indeed organized as a biofilm. Some species such as *Lactobacillus reuteri*, a commonly detected species in the intestinal microbiota (monoxenic mouse model; Frese et al., 2013), form *in vivo* mono-bacterial biofilms.

To address the question of the ability of *C. difficile* to organize in biofilms in the host gut, we specifically chose the *C. difficile* mono-associated mouse model which has three major advantages: first, this model allows the elucidation of an autonomous biofilm in the gut, as is observed *in vitro*; second, monoxenic mice are valuable experimental tools to investigate host–bacterial interactions in an environment devoid of competitive interactions and to study easily both the location of bacteria in the gut and the structure of biofilms, if any; third, this model allows *C. difficile* colonization without occurrence of clinical signs, and therefore allowed us to test our hypothesis *C. difficile* biofilms could be involved in (human) asymptomatic carriage and/or asymptomatic persistence. In contrast, in the conventional mouse model, colonization by *C. difficile* after strong antibiotherapy leads to gut lesions. In our model, however, no histological lesions were seen on gut sections of different strains (**Figure 3**).

As a first approach, we assessed the ability of four biofilm-producing strains (Pantaléon et al., 2015) to colonize germ-free mice. 630Δerm is a modified strain derived from a human clinical isolate, and the cwp84 mutant is further derived from this strain. The P30 strain has been isolated from poultry and may not been considered as a clinical strain. This strain has been shown to display an ecological advantage to colonize the mouse intestinal niche (Spigaglia et al., 2013). Thus, a human-origin, clinically relevant strain is R20291 isolated from an epidemic in United Kingdom, and belonging to the 027 lineage. This lineage is known to be associated particularly with a high rate of recurrence. All *in vivo* studies were carried out 7 days post-infection because we previously performed *in vivo* experiments in mono-associated mice 3 days post-infection but only few bacteria could be detected by confocal microscopy. Although the four strains displayed different abilities to form biofilm on polystyrene plates, they were able to achieve similar levels of colonization along the intestinal tract in our monoxenic mouse model. Therefore, we did not find any correlation between the ability of strains to form biofilm *in vitro* and their ability to associate with the mouse gut. Indeed, the poor biofilm-producing 630Δ*erm* strain colonizes the gut as well as the other strains. This lack of correlation is undoubtedly explained by the different environmental conditions prevailing *in vivo* and *in vitro*, in particular the nature of the surface which influences the biofilm formation. Of note, we observed that CD stimulates the maturation of villi and the production of mucus by goblet cells and to our knowledge, it is the first time that this feature is reported. However, several publications showed that commensal bacteria favor the development of mucus and vascular networks in the gut, and this may be correlated with maturation of villi (Stappenbeck et al., 2002).

The mucosa-associated bacteria were organized as a 3-D bacterial community, as observed by CLSM analysis in both the cecum and the colon of mice infected with the four strains. However, the organization of the bacterial communities in these parts of the mouse gut were different according to strains. Whereas the P30, the 630Δ*erm* and the *cwp84* mutant displayed mainly isolated bacteria, the R20291 strain formed numerous aggregates. These aggregates could correspond to microcolonies, which may result either from *in situ* multiplication of bacteria, or from mucosal reassociation of planktonic bacteria living in the luminal environment. This result is reminiscent with those obtained by Lawley et al. (2009) in a conventional mouse model: they observed mats of bacilli overlaying microvilli (Lawley et al., 2009), likely to be *C. difficile*.

One important objective of our study was to clarify the localization of tissue-associated bacteria with respect to the intestinal mucus layer. Many studies show that the inner layer of mucus is devoid of bacteria, and that the outer layer is associated with bacteria (Hansson and Johansson, 2010; Johansson et al., 2011). In accordance with Semenyuk et al. (2015), we found few bacteria localized in the mucus (see **Figure 3**). However, we also visualized several *C. difficile* vegetative cells localized outside the outer layer of mucus (**Figures 3–5**). This discrepancy may be related to the different animal models used. Indeed, the microbiota influence the composition and physicochemical properties of the mucus, and may result in a potentially modified penetration of bacteria in conventional as well as monoxenic mice (Johansson et al., 2015).

In our model, and following efficient rinsing, bacteria were still entrapped in 3-D structures supported at the mucus layer. Interestingly, in addition to bacterial cells, we also observed diffuse labeling with SYTO^®^ 9 reminiscent of extracellular DNA, a matrix component found in *in vitro C. difficile* biofilms (Đapa et al., 2013; Semenyuk et al., 2014). These structures were observed for the three strains tested.

To further analyze the possibility that tissue-associated *C. difficile* cells were encased in an extrapolymeric matrix, we labeled gut sections of a mouse infected with the R20291 with antibodies recognizing the cell wall-associated polysaccharide II of *C. difficile*, another component of the matrix of *in vitro* biofilm (Đapa et al., 2013; Semenyuk et al., 2014). We showed that the bacteria overlaying the mucus layer are surrounded by a large amount of PS-II. In planktonic mode, PS-II is the main surface-associated polysaccharide and it is ubiquitous in all *C. difficile* strains (Danieli et al., 2011; Chu et al., 2016; Monteiro, 2016). Nevertheless, the intensity and distribution of labeling as compared to the distribution of bacteria in the same location are in accordance with extracellular PS-II entrapping the bacteria. In addition, bacteria present in the mucus layer (Supplementary Figure S3C) are not labeled by the anti-PS-II in the same conditions. As those structures were not observed in gut section of the axenic mice, we hypothesized that bacteria overlaying the mucus layer are organized as a biofilm entrapped in a glycan matrix composed at least of PS-II and possibly also DNA. Of note, these structures seem to be smaller in spatial extension than *in vitro* biofilms, but discrepancies between sizes of *in vitro* and *in vivo* biofilm have been already observed (Bjarnsholt et al., 2013). Indeed, the *in vivo* model is a dynamic model subject to various environmental stresses such as intestinal peristalsis, continuous flow, passage of bolus, in contrast to the static *in vitro* model which provides a stable environment but with a decreased nutrient availability over time (Lebeaux et al., 2013). This could undoubtedly contribute to the small size of *in vivo* biofilm structures. As revealed by immunochemistry (**Figures 3–5**), biofilm structures were present as small islets irregularly distributed over the mucosa, and this is relevant with the large-scale observations made by CLSM on the heterogeneous distribution of bacteria over the gut mouse tissues. This could be explained either by (i) the detachment of mature biofilm, (ii) removal of biofilm due to the natural mucus renewal (Frese et al., 2013) or (iii) by a specific interaction with an underlying intestinal tissue (Sommer et al., 2015).

To our knowledge, this report is the first description of the development *in vivo* of a *stricto sensu* biofilm of *C. difficile*. More investigations are now necessary to validate this biofilm-structure in other clinically relevant *C. difficile* strains, and to elucidate its putative role in the colonization and persistence of *C. difficile*.

## Author Contributions

Conceived and designed the experiments: SB, CJ, and GV. Performed the experiments: AS, SB, SH, FG, and VN. Analyzed the data: AS, SB, CJ, and GV. Contributed reagents/materials/analysis tools: GV. Wrote the paper: AS, SB, CJ, and GV.

## Conflict of Interest Statement

The authors declare that the research was conducted in the absence of any commercial or financial relationships that could be construed as a potential conflict of interest.
