# Supplementary material for: Biofilm Structures in a Mono-Associated Mouse Model of Clostridium difficile Infection
Source: Front Microbiol. 2017 Oct 25;8:2086. doi: 10.3389/fmicb.2017.02086 (PMC5661025; doi:10.3389/fmicb.2017.02086)
Supplement: Supplementary file 1 [file Data_Sheet_1.PDF]

Supporting information

FIGURE S1. The four strains tested display a significant difference in biofilm producing ability.

(**A**) Crystal violet staining of biofilm. Strains were classified into three categories: one high-biofilm former (*cwp84* mutant) (mean absorbance > 15), two moderate-biofilm formers (R20291 and P30) (5 < mean absorbance < 15) and one low-biofilm former (mean absorbance < 5) (630Δ*erm*). (**B**) Quantitation of viable vegetative cells in the biofilm. (**C**) Z-stack images from confocal laser scanning microscopy of *in vitro* biofilm obtained from the four strains tested. (**D**) Mean biofilm thickness obtained from CLSM images. The data represent the average of at least four independent assays. Statistical analyze was performed by Mann-Whitney test. * 0.025 < p < 0,05, ** 0,0125 < p < 0,025, *** p < 0,0125

FIGURE S2. Bacteria overlaying the gut mucosa
C. difficile R 20291 overlying the gut mucosa in cecum (**A** and **B**), and in colon (**C** and **D**). Bacterial and eukaryotic DNA are stained by Hoechst (33342). Scale bar is 50 µm.

FIGURE S3. Bacteria localized in the mucus layer are not embedded in a PS-II matrix in the cecum and colon of R20291.
We compared serial sections **(C, D)** that were stained differently. Mucus and PS-II are stained in red and DNA (bacteria and epithelial tissue, extracellular DNA) are stained in blue. Scale bar is 100 µm.

FIGURE S4. **Production of mucus in presence of *C. difficile*.**
Immunodetection of the mucus was performed with anti-Muc2 antibodies. (**A**) Section of cecum and colon after infection by the *C. difficile* R20291 strain. Mucus is stained in brown. The right panel is the enlargement of the yellow boxed portion of the image: “o” indicates the outer mucus layer, and “i” the inner mucus. Arrow indicates position of the goblet cells. Scale bar is 50 µm. (**B**) Numeration of Muc2-containing goblet cells per villi in cecum and colon. Error bars indicate the standard deviation of the means. Statistical analyses were performed using the Mann-Whitney test.
