# Supplementary figures and images for: Biofilm Structures in a Mono-Associated Mouse Model of Clostridium difficile Infection
Source: Front Microbiol. 2017 Oct 25;8:2086. doi: 10.3389/fmicb.2017.02086 (PMC5661025; doi:10.3389/fmicb.2017.02086)

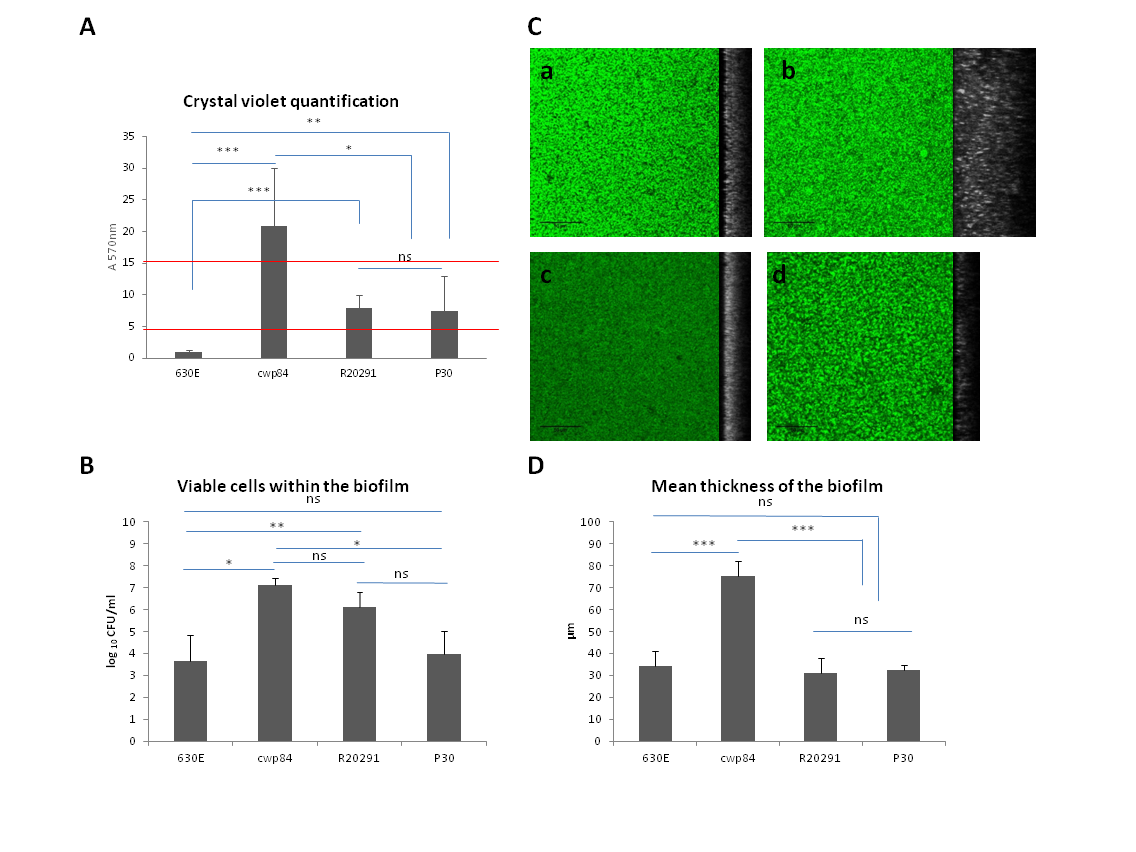

Supplement: Supplementary file 2 [file Image_1.TIF]

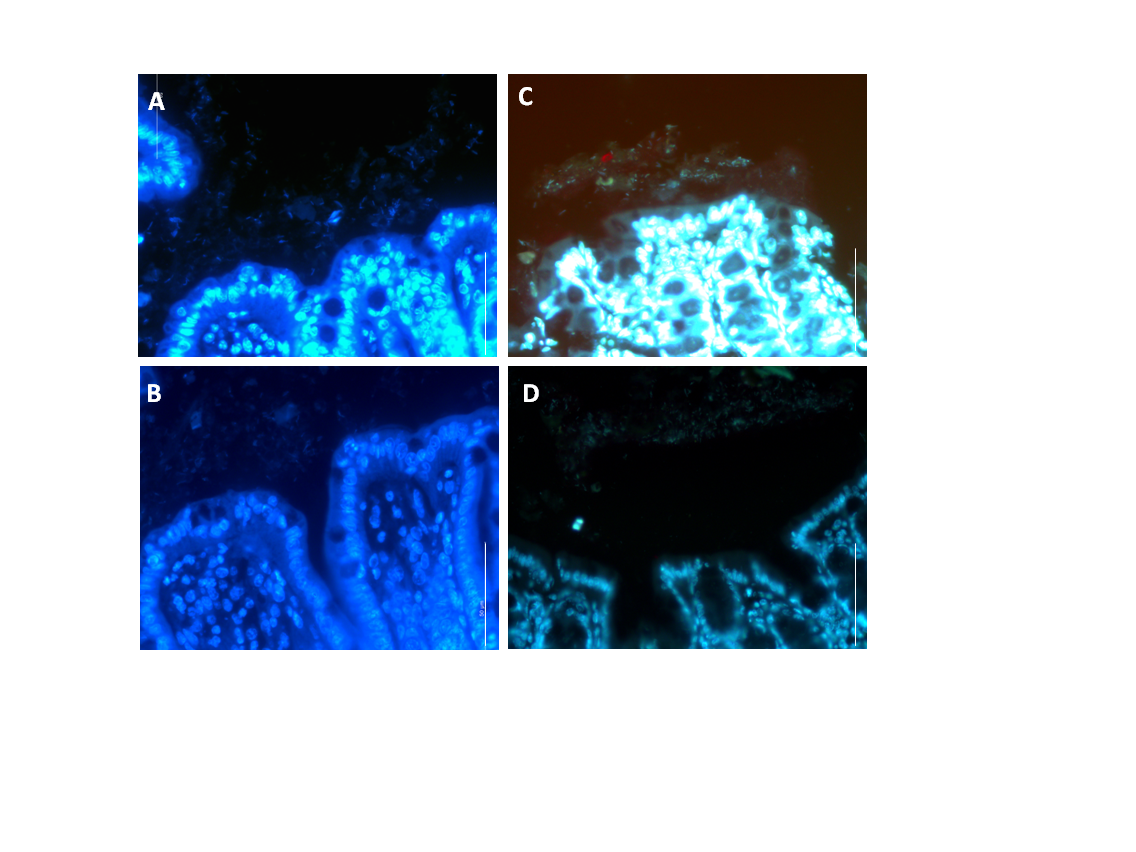

Supplement: Supplementary file 3 [file Image_2.TIF]

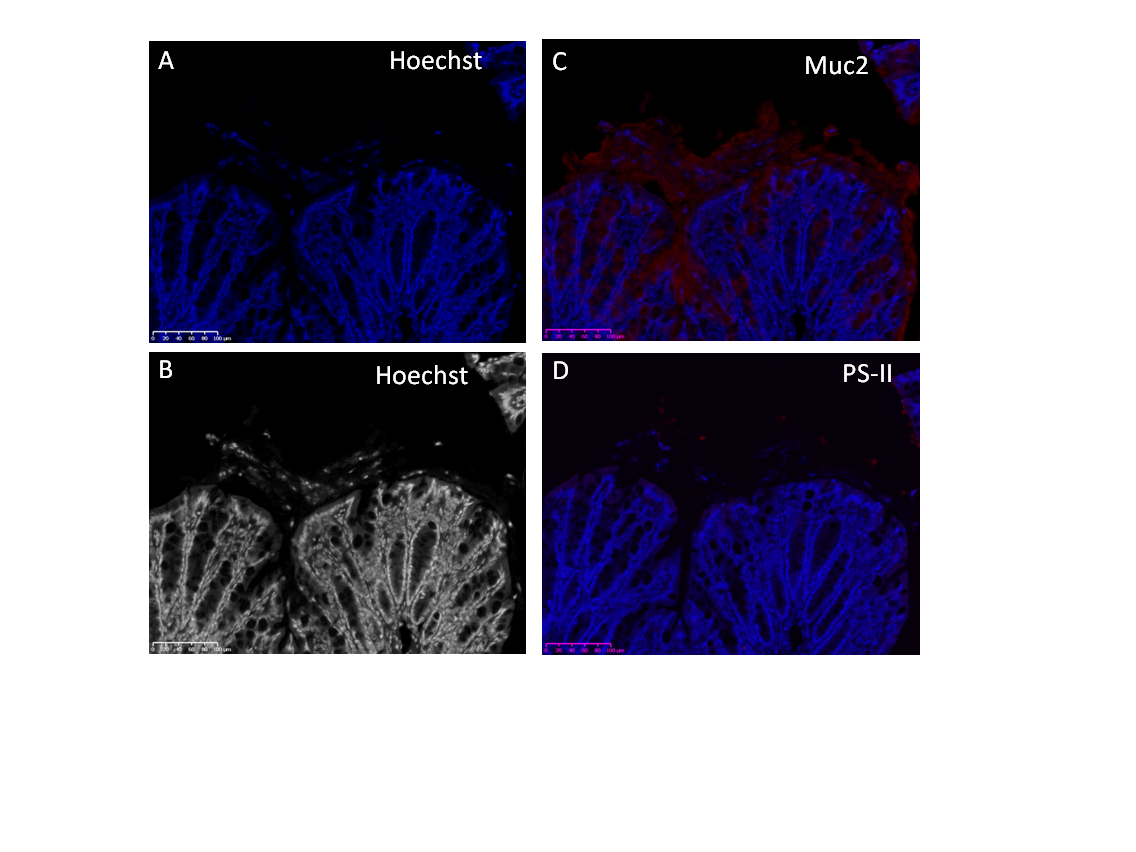

Supplement: Supplementary file 4 [file Image_3.TIF]

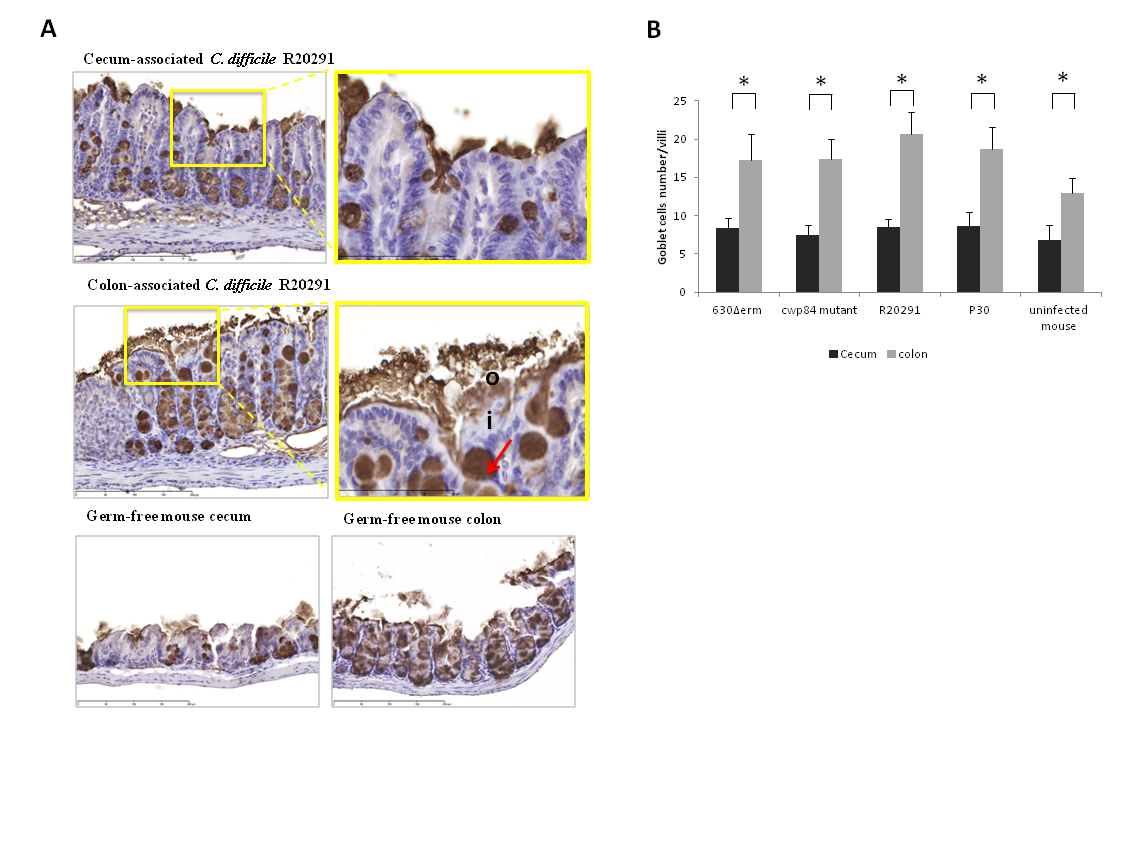

Supplement: Supplementary file 5 [file Image_4.TIF]
